# Salivary FRAP as A Marker of Chronic Kidney Disease Progression in Children

**DOI:** 10.3390/antiox8090409

**Published:** 2019-09-18

**Authors:** Mateusz Maciejczyk, Julita Szulimowska, Katarzyna Taranta-Janusz, Katarzyna Werbel, Anna Wasilewska, Anna Zalewska

**Affiliations:** 1 Department of Hygiene, Medical University of Bialystok, 15-233 Bialystok, Poland; 2 Department of Pedodontics, Medical University of Bialystok, 15-274 Bialystok, Poland; szulimowska.julita@gmail.com; 3 Department of Pediatrics and Nephrology, Medical University of Bialystok, 15-274 Bialystok, Poland; katarzyna.taranta@wp.pl (K.T.-J.); katarzynawerbel@gmail.com (K.W.); annwasil@interia.pl (A.W.); 4 Department of Conservative Dentistry, Medical University of Bialystok, 15-274 Bialystok, Poland; azalewska426@gmail.com

**Keywords:** chronic kidney disease, salivary biomarkers, redox homeostasis, salivary FRAP

## Abstract

Chronic kidney disease (CKD) is one of the most common modern-age diseases in children. Kidney failure does not reveal any symptoms for a long time; therefore, new biomarkers are sought, preferably those reflecting an early stage of CKD. The aim of our study was to evaluate total antioxidant potential as a biomarker differentiating the degree of CKD advancement. The study included 30 children with CKD and a control group matched by age and gender. Non-stimulated saliva (NWS), stimulated saliva (SWS), plasma and urine were used as study material. Total antioxidant potential was determined spectrophotometrically using the FRAP method (ferric ion reducing antioxidant parameter) by measuring total FRAP and uric acid (UA)-independent FRAP (FRAP-UA). We demonstrated that total FRAP, FRAP-UA and UA were significantly higher in stimulated saliva, as well as urine of CKD patients compared to the controls. These biomarkers increase with the progression of chronic kidney disease and their concentration in SWS reflects their content in urine. Interestingly, salivary FRAP and uric acid clearly differentiate between various stages of CKD as well as between healthy and ill children. Special attention should be paid to total FRAP which—measured in SWS—distinguishes patients with mildly to moderately decreased kidney function from those with severe renal impairment (AUC = 1, sensitivity = 100%, specificity = 100%). Although salivary FRAP may be a potential CKD biomarker in children, further studies are needed in a larger group of patients.

## 1. Introduction

Chronic kidney disease (CKD) is a syndrome giving a multitude of symptoms and resulting from a permanent reduction in the number of nephrons, or their permanent damage [1]. CKD is characterized by progressive decrease of glomerular filtration rate (GFR) and is one of the most common lifestyle diseases not only in adults, but also in children. Although the amount of accurate epidemiological data on the prevalence of CKD in children is limited, mortality in this group of patients is very considerable—about 30 times higher than the expected mortality rate at a given age [2,3]. According to the current guidelines [4], CKD is diagnosed based on structural or functional impairment of renal function and/or reduction of GFR to less than 60 mL/min/1.73 m^2^, lasting more than three months. Other CKD indices include: albuminuria, abnormal urine sediment, electrolyte changes and deviations in imaging and kidney biopsy [5]. However, diagnosing CKD in children and adolescents may entail considerable difficulties. First, GFR in young people changes with age. Second, in lean individuals with low muscle mass (i.e., children and the elderly), creatinine levels may be normal even with significant deterioration in kidney function [2,5]. Therefore, there is still a need for new biomarkers that will reflect kidney function more precisely.

It is assumed that oxidative stress plays a key role in CKD aetiology [6,7,8]. It has been demonstrated that overproduction of free radicals in CKD is caused by increased activity of the renin-angiotensin-aldosterone system (RAAS), inflammation, and disturbed mitochondrial functioning. Indeed, angiotensin II increases the proinflammatory NF-κB signalling pathway, which boosts not only cytokine production but also the production of reactive oxygen species (ROS). Numerous studies have demonstrated that oxidative stress leads to the oxidation of proteins and lipids in the kidney parenchyma, resulting in progressive dysfunction of this organ [6,7,8]. It is, therefore, not surprising that the use of oxidative stress biomarkers in differential diagnosis of CKD patients is postulated [9,10,11,12].

In modern laboratory diagnostics there is a growing interest in alternative biological materials to be collected without specialist equipment and supervision of medical personnel. This may contribute to the reduction of patients’ anxiety, encourage people to monitor their health more frequently, and enable early diagnosis of the disease. Saliva serves as a non-invasive, and at the same time non-infectious, diagnostic material [13]. Although this fluid consists of over 95% water, it is also a rich source of antioxidants. These include salivary peroxidase, catalase, superoxide dismutase, uric acid (UA), glutathione and thioredoxin, of which 70–80% of the antioxidant activity is served by UA [14,15]. Interestingly, salivary antioxidants are increasingly popular in the diagnosis of a number of systemic diseases. Clinical usefulness of salivary redox biomarkers has been confirmed in patients with diabetes [16,17], obesity [18,19], neurodegenerative diseases [20,21] as well as in CKD patients [11,22].

As we have demonstrated in the previous studies [11], CKD is accompanied by disturbances of enzymatic and non-enzymatic antioxidant systems and intensified oxidative damage to proteins and lipids, not only in plasma but also saliva. Generally, salivary oxidative stress indices correlate with their content in blood and may serve as potential diagnostic biomarkers of CKD in children. However, none of the parameters evaluated so far has differentiated between various stages of chronic kidney disease. Due to the lack of commonly available diagnostic tools and the fact that CKD does not give any clinical manifestations of kidney disease in the early stages, the disease is often diagnosed when the deterioration in kidney function cannot be slowed. Therefore, the aim of our study was to search for a non-invasive biomarker of chronic kidney disease progression in children. For this purpose, we decided to evaluate the total antioxidant potential, using the FRAP (ferric ion reducing antioxidant) method, in non-stimulated and stimulated saliva, plasma and urine of patients with CKD and healthy controls. Since uric acid strongly affects the value of total antioxidant capacity, we measured both total FRAP and UA-independent FRAP (FRAP-UA) [23].

## 2. Material and Methods

### 2.1. Ethical Issues

The research project was approved by the Local Bioethical Committee in Bialystok (permission number R-I-002/43/2018). It was conducted in accordance with the Declaration of Helsinki determining ethical principles for medical research involving humans. All subjects and/or their legal guardians consented in writing to participate in the experiment.

### 2.2. Patients

The study included 30 CKD children with normal body weight, treated at the Department of Pediatrics and Nephrology of the Medical University of Bialystok Children’s Clinical Hospital. The patients’ condition was assessed based on medical history, physical examination, and laboratory and imaging results. The causes of CKD were: glomerulopathy (30%), urological abnormalities (23.3%), nephropathy (16.7%), kidney dysplasia (16.7%) and unknown aetiology (13.3%).

CKD was defined according to the Kidney Disease Improving Global Outcomes (KDIGO) criteria [4] based on different GFR distribution: Stage 1: >90 mL/min; Stage 2: 60–89 mL/min; Stage 3: 30–59 mL/min; Stage 4: 15–29 mL/min; and Stage 5: <15 mL/min. The estimated glomerular filtration rate (eGFR) was calculated using the updated Schwartz formula: eGFR (mL/min/1.73 m^2^) = 0.413 × (height in cm/sCr) [24]. Kidney function impairment was also assessed based on the levels of serum creatinine (sCr), serum urea, as well as urine protein (albuminuria, proteinuria).

Blood pressure was measured using either a manual auscultatory or an automatic oscillometric device after the patient had rested for 5 min in a sitting position. The average values of the second and third measurements were used for subsequent analyses, based on a decision concerning hypertension. Hypertension was defined as the average value of the systolic and/or diastolic blood pleasure of ≥ 95th percentile for age, gender, and height [25].

From the moment of positive CKD diagnosis, all patients were on renal diet low in sodium and/or phosphorous and/or protein, depending on a patient’s condition and CKD stage [26].

The control group consisted of generally healthy children with normal body weight who attended follow-up visits at the Children’s Dental Clinic at the Specialist Dental Clinic of the Medical University of Bialystok. The control was matched by age and gender to the study group.

The exclusion criterion in both groups was the occurrence of systemic, metabolic, autoimmune, neoplastic, and infectious diseases, as well as diseases of the digestive tract, thyroid and lungs. Patients taking glucocorticosteroids, non-steroidal anti-inflammatory drugs (NSAIDs), hormones, antibiotics, vitamins and dietary supplements for at least three months prior to the study, as well as cigarette smokers and people with poor oral hygiene and/or gum inflammation, were excluded from the study (see: dental examination).

Detailed clinical characteristics of patients and the controls are presented in Table 1.

### 2.3. Saliva Collection

Total mixed non-stimulated saliva (NWS) and stimulated saliva (SWS) were used for assays. Saliva was collected between 7:30 a.m. and 9:00 a.m. after an overnight rest from patients who had refrained from increased physical activity for the preceding 24 h. Patients were advised not to consume any food or drink other than clean water at least 2 h before saliva collection and not to perform any oral hygiene procedures (brushing, chewing gum, etc.). Furthermore, subjects from the study as well as the control group had not taken any medicines at least 8 h prior to saliva collection.

Saliva was collected via the spitting method, each time in the same, child-friendly room (after at least a 5-min adaptation period). Before the collection, the oral cavity was rinsed twice with distilled water at room temperature. Saliva was collected in a sitting position with the head slightly inclined downwards, and with limited movements of the face and lips. Saliva was spat into a sterile tube placed in a container with ice, and the samples collected during the first minute were discarded. NWS was collected for 15 min. SWS was collected similarly to NWS and its secretion was stimulated by sprinkling the tip of the tongue with 10 µL of 2% citric acid every 30 s. SWS was collected for 5 min. No blood contamination was observed in any of the samples. Immediately after collection, saliva was centrifuged (20 min, 4 °C, 3000× *g*; MPW 351, MPW Med. Instruments, Warsaw, Poland). In order to protect the samples from oxidation processes, butylated hydroxytoluene (BHT, Sigma-Aldrich, Saint Louis, USA) was added to the obtained supernatants in the amount of 10 μL 0.5 M BHT in acetonitrile (ACN)/1 mL of saliva [27]. The samples were frozen at −80 °C and stored for no more than six months.

Immediately after collection, the volume of saliva was measured using a calibrated pipette with the accuracy of 100 µL. The minute flow of NWS and SWS was calculated by dividing the saliva volume by the time necessary for its secretion (mL/min) [27].

### 2.4. Dental Examination

Right after saliva collection, a clinical dental examination was performed in every patient according to the World Health Organization criteria [28]. A mirror, an explorer and a periodontal probe were used in artificial lighting. The incidence of caries was determined by the DMFT (decayed, missing, filled teeth) index which is the sum of teeth with caries (D), teeth extracted as a result of caries (M) and teeth filled because of caries (F). DMFT was also calculated for deciduous teeth (dmft). The evaluation of oral hygiene was performed based on API (approximal plaque index) according to Lange. API determines the percentage of tooth surface with plaque. To assess the condition of gums, we used SBI (Sulcus Bleeding Index) according to Mühlemann and Son, and GI (Gingival Index) according to Löe and Silness. The SBI shows the intensity of bleeding from the gingival sulcus after probing, while GI criteria include qualitative changes in the gingiva [28].

Persons with poor oral hygiene (API > 20) and gum inflammation (SBI > 0.5, GI > 0.5) were excluded from the experiment. All the dental examinations were performed by the same dentist (J. S.). In 10 patients we assessed the inter-rater agreements between the examiner (J. S.) and another experienced dentist (A. Z.). The reliability for DMFT was r = 0.96; dmft = 0.92; API was r = 1.0; SBI was r = 0.98; and GI was r = 0.96.

### 2.5. Plasma and Urine Collection

Whole blood was collected between 7 a.m. and 8 a.m. in a sitting position, from fasting patients after an overnight rest. S-Monovette^®^ K3 EDTA blood collection system (Sarstedt, Nümbrecht, Germany) was used, and samples were centrifuged immediately (1500× *g*; 4 °C, 10 min). No haemolysis was found in any sample, and the supernatant fluid (plasma) was preserved for further assays.

Urine was collected into a sterile container from the middle stream, after an overnight resting period and morning crotch hygiene procedures. Within 1 h of collections, the samples were delivered to the laboratory and centrifuged immediately (400× *g*, 4 °C, 10 min).

Similar to saliva, BHT (10 μL 0.5 M BHT in ACN/1 mL of the sample) was added to plasma and urine samples that were then frozen at −80 °C until assayed [27].

### 2.6. Total Protein Assay

The total protein content was determined via the bicinchoninic acid (BCA) method using a commercial kit (Thermo Scientific PIERCE BCA Protein Assay (Rockford, IL, USA)). Bovine serum albumin (BSA) was used as a standard.

### 2.7. FRAP Assay

The total antioxidant potential of saliva, plasma and urine was determined spectrophotometrically using FRAP [23]. This method is based on the reduction of Fe^3+^ ions in the form of a complex with 2,4,6-Tri(2-pyridyl)-s-triazine (TPTZ) to Fe^2+^ ions. The TPTZ-Fe^2+^ complex has intense colour with a maximum absorption at 593 nm wavelength. The colour intensity is directly proportional to the concentration of Fe^2+^ ions. Since uric acid strongly affects the value of total antioxidant potential, both total FRAP and UA-independent FRAP (FRAP-UA) were measured after the addition of uricase. UA concentration was calculated from the difference between total FRAP and FRAP-UA, whereas uric acid level in the total antioxidant potential of saliva, plasma, and urine was expressed as:

(1) $$\Delta \mathrm{FRAP}=\frac{\left[\mathrm{total}\;\mathrm{FRAP}\right]-\left[\mathrm{FRAP}\;\mathrm{UA}\right]}{\left[\mathrm{total}\;\mathrm{FRAP}\right]}\times 100\%.$$

All reagents were purchased from Sigma-Aldrich (Nümbrecht, Germany and/or Saint Louis, MO, USA). 60 μL of PBS (pH 7.4) or 60 μL of uricase (10 U/mL) were added to 200 μL of the sample, then mixed thoroughly and incubated for 20 min at 25 °C. Next, 100 μL of the sample was taken, and 3 mL of freshly prepared FRAP reagent was added to the sample by mixing acetate buffer (300 mM/L, pH 3.6), TPTZ (10 mM/L in 40 mM/L HCl) and FeCl_3_ (20 mM/L) at a volume ratio of 10:1:1. All the said substances were mixed, and after 8 min we measured the absorbance changes at 593 nm wavelength as compared to the blank test. The absorbance was measured with Infinite M200 PRO Multimode Microplate Reader from Tecan. The standard curve was prepared by dissolving uric acid (84 mg) and lithium carbonate (60 mg) in deionized water (30 mL) at 60 °C. The resulting solution was diluted with deionized water to 100 mL (5 mM/L stock UA standard solution). The solution was protected from light, and used directly to prepare the diluted solutions of the standards [23].

All measurements were performed in triplicate samples. The results were standardised to mg of total protein.

### 2.8. Statistical Analysis

Statistical analysis was performed using the GraphPad Prism 7 (GraphPad Software, La Jolla, USA). The Shapiro–Wilk test was used to determine the normality of distribution, and comparisons were made with the one-way ANOVA variance analysis and the Tukey test (comparison of several groups) or the unpaired Student’s *t*-test (comparisons between two groups). Multiplicity adjusted *p* values were also calculated. The correlation of the obtained results was assessed using the Pearson correlation coefficient. The zero hypotheses were falsified at the materiality level *p* = 0.05. The results (expressed as an arithmetic mean ± SEM) are presented in the figures and tables included herein. ROC (receiver operating characteristic) analysis was used to assess the diagnostic usefulness of the assayed biomarkers. Diagnostic value of salivary FRAP was evaluated between healthy and CKD patients as well as between patients with weak/moderate renal failure (stages 1–3) and those with severe CKD (stages 4–5). The number of patients was selected based on the previous pilot study, with the value of 0.9 assumed as the test strength.

## 3. Results

### 3.1. Salivary Gland Function and Dental Examination

The content of total protein (TP) was significantly lower in NWS of children at stage 1 (*p* = 0.003), 2 (*p* = 0.01), 3 (*p* = 0.008) and 5 (*p* = 0.007) of chronic kidney disease, as well as in all patients with CKD (*p* < 0.001) compared to the controls. The same changes were observed at all stages of CKD in stimulated saliva (*p* < 0.001 versus the controls) (Table 2).

The minute flow of non-stimulated saliva was significantly lower in all groups of children with CKD compared to the control (*p* < 0.001). The rate of SWS secretion was lower in children at stage 1, 2, 3 and 4 of CKD, as well as in all patients with chronic kidney disease compared to healthy controls (*p* < 0.001) (Table 2).

We have not observed any significant differences in the incidence of caries, oral hygiene, or the condition of the gums between the studied groups (Table 2).

### 3.2. Total FRAP

**Figure 1:**
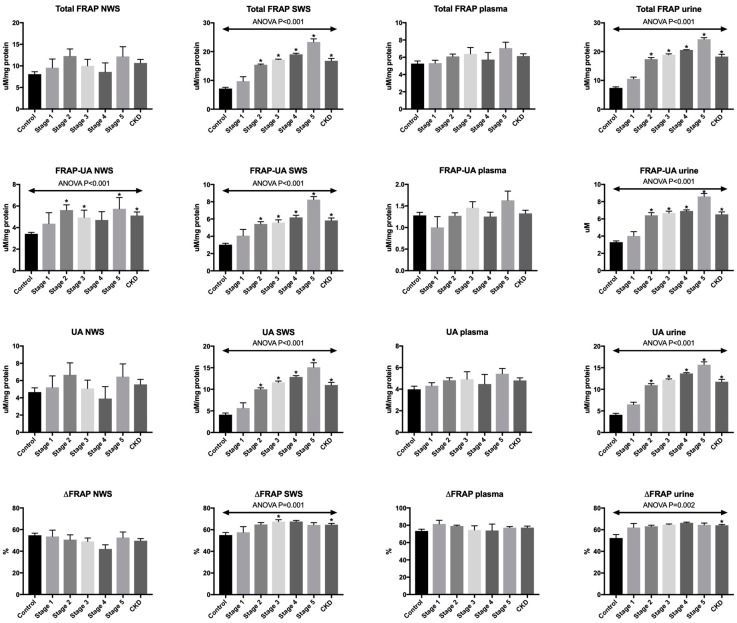
Total antioxidant capacity measured by FRAP in saliva, plasma and urine of children at different stages of chronic kidney disease (CKD) and healthy controls. FRAP—ferric ion reducing antioxidant parameter; FRAP-UA—UA-independent FRAP; NWS—non-stimulated whole saliva; SWS—stimulated whole saliva; UA—uric acid. * *p* < 0.05 vs. the control group.

Total antioxidant potential of NWS and plasma expressed as total FRAP did not differ significantly between the studied groups. However, in SWS and urine total FRAP levels were considerably higher at stage 2, 3, 4 and 5 of chronic kidney disease, as well as in all CKD patients compared to the controls (*p* < 0.001) (Figure 1).

### 3.3. FRAP-UA

UA-independent FRAP (FRAP-UA) was significantly higher in NWS of children at stage 2 (*p* = 0.01) and 5 (*p* = 0.04) of CKD compared to healthy controls, similarly to all CKD patients (*p* = 0.001) vs. the control. The level of UA-independent FRAP was also higher in SWS and urine of children at stages 2–5 of CKD and in all patients compared to healthy subjects (*p* < 0.001) (Figure 1).

### 3.4. UA

Uric acid concentration calculated as the difference between total FRAP and UA-independent FRAP did not differ significantly in NWS and plasma of the both groups. However, in SWS and urine, the level of UA was considerably higher at stages 2–5 of CKD, as well as in all CKD patients compared to the controls (*p* < 0.001) (Figure 1).

### 3.5. ΔFRAP

The share of uric acid in the total antioxidant potential, calculated as ΔFRAP, did not differ both in NWS and plasma of all the analysed groups. In stimulated saliva, ΔFRAP was significantly higher at stage 3 of CKD (*p* = 0.03) and in the group of all patients with chronic kidney disease versus healthy controls (*p* = 0.003). This parameter was considerably higher in urine of only the group of all CKD patients compared to the controls (*p* = 0.001) (Figure 1).

### 3.6. ROC Analysis

All the biomarkers evaluated in stimulated saliva and urine significantly differentiated patients from healthy subjects. High diagnostic value in distinguishing children with chronic kidney disease from the controls was also confirmed for total FRAP and FRAP-UA in non-stimulated saliva, and total FRAP and UA in plasma (Table 3).

**Figure 2:**
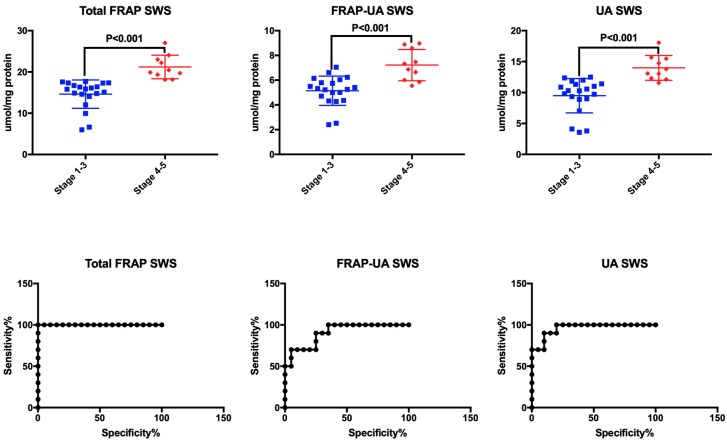
Receiver operating characteristic (ROC) analysis of total antioxidant capacity measured by FRAP in stimulated saliva of children at stage 1–3 and stage 4–5 of chronic kidney disease (CKD). FRAP—ferric ion reducing antioxidant parameter; FRAP-UA—UA-independent FRAP; SWS—stimulated whole saliva; UA—uric acid.

ROC analysis demonstrated that at high sensitivity and specificity, total FRAP, FRAP-UA and UA in stimulated saliva significantly differentiated children at stage 1–3 of CKD from patients at stage 4 or 5 of the disease. Interestingly, the level of total FRAP > 17.92 μM/mg protein in stimulated saliva differentiated between patients with weak/moderate renal failure and those with severe CKD (AUC = 1, sensitivity = 100%, specificity = 100%). All urinary biomarkers significantly distinguished children at stage 1–3 of CKD from those at stage 4–5 of renal failure (Table 4, Figure 2).

### 3.7. Correlations

The correlations between redox biomarkers evaluated in saliva, plasma and urine of patients with chronic kidney disease are presented in Table 5. Interestingly, the total FRAP level in SWS revealed significantly positive correlation with its content in urine, and similar results were observed for the concentration of uric acid (Table 5).

The correlations between salivary antioxidant potential and clinical parameters for patients with CKD are presented in Table 6. The concentrations of total FRAP, FRAP-UA and UA in SWS correlated negatively with the value of eGFR, and positively with serum creatinine and urea. Positive correlations between FRAP in SWS and proteinuria and albuminuria are also noteworthy. A similar correlation was found for uric acid in SWS. A negative correlation between total FRAP, FRAP-UA and UA in SWS and the levels of haemoglobin and haematocrit in serum of CKD children was also found. These biomarkers also positively correlated with the concentration of iron and ferritin (Table 6).

## 4. Discussion

Chronic kidney disease is inseparably connected with oxidative stress [6,7,8]. In the course of CKD, enzymatic and non-enzymatic disturbances of antioxidant systems and severe oxidative damage occur, resulting in progressive renal failure [6,7,8].

All antioxidants basically fulfil the same function: they impede the formation of ROS and interrupt free radical reactions, preventing the interaction of oxidants with cellular components. However, it is difficult to draw conclusions about redox homeostasis solely based on the assessment of individual antioxidants. A much better parameter is total antioxidant potential which determines the total capacity of our biological system to scavenge free radicals. It is believed that total antioxidant potential includes interactions between all antioxidants [23,29]. In our study, the total antioxidant activity was evaluated using FRAP (ferric ion reducing antioxidant parameter). The levels of total FRAP, UA-independent FRAP (FRAP-UA), uric acid, and ΔFRAP were significantly higher in stimulated saliva and urine of CKD patients than in healthy controls.

A rich source of antioxidants is saliva [15,30]. This fluid is produced by large salivary glands (submandibular, parotid and sublingual) as well as numerous smaller salivary glands scattered throughout the oral cavity. Most of it is produced by submandibular glands (about 70% of its overall volume), while 25% of saliva is secreted from parotid glands. While stimulated (with food or chemical stimuli), parotid glands prevail over submandibular glands in terms of the amount of produced saliva [21]. Considering the fact that the main source of antioxidants in the oral cavity is the parotid gland [31,32], it is not surprising that higher levels of FRAP, FRAP-UA and UA were observed in stimulated saliva compared to NWS. It is worth recalling that parotid glands are highly aerobic organs equipped with a very effective mitochondrial antioxidant system [33,34]. Under oxidative stress conditions, the antioxidative barrier may be strengthened, which was confirmed by the results of our study. Indeed, the increase in FRAP, FRAP-UA and UA contents in the saliva of children with CKD compared to the controls suggests an adaptive response of the body to increased ROS production. It was demonstrated that the overproduction of free radicals in CKD is caused by overactivation of RAAS, which impairs the function of mitochondria and increases the activity of NOX (NADPH oxidase)—the primary source of ROS in the cell [6,7]. Angiotensin II also increases the expression of vascular adhesion molecule-1 (VCAM-1) and intercellular adhesion molecule-1 (ICAM-1), which activates the NF-κB pathway and further intensifies oxidative stress [6,7,8]. In our earlier study, we also observed enhanced antioxidant defence in the saliva of CKD patients, expressed as increased activity of salivary peroxidase and superoxide dismutase, as well as in the concentration of salivary uric acid and albumin [11]. Strengthened antioxidant barriers in the saliva of children with chronic kidney disease is not surprising, especially since the oral cavity is the only place in the body exposed to numerous pro-oxidative factors, such as diet, medicines, microorganisms, and air pollution [30]. Negative correlation between the age of CKD diagnosis and total FRAP and UA concentrations in stimulated saliva is also noteworthy, as it proves an increase of salivary redox disorders with the disease duration.

Laboratory evaluation of kidney function is an important element of CKD diagnostics. Progressive renal failure reveals no symptoms for a long time, and early stages of CKD may be latent in both clinical and laboratory tests. Therefore, new biomarkers are constantly being sought to reflect particularly the initial stage of kidney damage [2,5]. Although creatinine and urea assays in serum are the simplest and most commonly applied method of evaluating kidney function, frequent blood collection for testing is not an indifferent procedure for patients (especially children). Therefore, the use of saliva is gaining popularity in laboratory medical diagnostics. The advantages of saliva include its easy, non-invasive and stress-free collection. Saliva is also relatively durable and can be collected several times a day [13,27].

The term ‘laboratory biomarker’ refers to an endogenous substance which, measured by validated and reproducible methods, correlates with the pathological process, is characterized by high sensitivity and specificity, and at the same time is easy to interpret [13]. The results of our study indicated the possibility of using FRAP in the differential diagnosis of patients with chronic kidney disease. The total antioxidant potential (total FRAP and FRAP-UA) and uric acid concentration were significantly higher in stimulated saliva of children with CKD compared to healthy controls. The levels of these biomarkers increase with the progression of chronic kidney disease and their concentration in SWS reflects their content in urine. Interestingly, salivary FRAP (both total FRAP and FRAP-UA) and uric acid clearly distinguish between different stages of CKD as well as between healthy individuals and CKD patients.

GFR (glomerular filtration rate) is the gold standard in CKD diagnostics [4]. However, at the early stages of the disease, it is necessary to conduct either laboratory or imaging tests to find additional exponents of kidney damage [2,5]. In our study, total FRAP, FRAP-UA and UA concentrations in SWS correlated negatively with eGFR (r = −0.83, r = −0.72, r = −0.79, respectively) and positively with serum creatinine (r = 0.78, r = 0.79, r = 0.69, respectively) and urea (r = 0.73, r = 0.70, r = 0.67, respectively). This indicates the relation of these biomarkers to the progression of chronic kidney disease, which is also confirmed by the positive correlation between salivary FRAP and proteinuria and albuminuria, as well as between uric acid in SWS and protein content in urine. Albuminuria is the earliest renal damage indicator among those normally determined in clinical practice [35]. Increased excretion of albumin in urine indicates the beginning of evident proteinuria as well as progression to end stage renal disease and cardiovascular disorders. It is believed that patients with high levels of proteinuria are more prone to develop chronic impairment of kidney parenchyma than a comparable group with low protein excretion [5,35]. Not without significance is also the outstandingly positive correlation between the concentration of the assayed parameters in SWS and urine (total FRAP, r = 0.94; FRAP-UA, r = 0.65; UA, r = 0.93). Thus, salivary FRAP and uric acid can be useful for monitoring CKD progression.

Renal anaemia is a significant clinical problem in children with CKD [4]. Its primary cause is erythropoietin deficiency resulting from weakened response of periurethral cells to hypoxia and iron deficiency. It is assumed that anaemia occurs at the early stages of CKD and intensifies with the disease progression [2,5]. Thus, it leads to numerous cardiovascular complications and significantly increases the mortality rate of patients. The results of our study indicate that salivary antioxidant potential measured by FRAP may be an exponent of erythropoietic disorders in CKD patients. It is evidenced by the negative correlation between total FRAP, FRAP-UA and UA in SWS and haemoglobin and haematocrit levels in serum of children with CKD. Salivary redox biomarkers also positively correlate with iron and ferritin serum concentrations, which is not surprising as most patients had iron supplementation prescribed.

The diagnostic value of the assessed redox biomarkers was also confirmed by ROC analysis. With high sensitivity and specificity, total FRAP, FRAP-UA and UA in stimulated saliva significantly differentiated healthy subjects from CKD patients, as well as children at stages 1–3 from those at stages 4–5 of the disease progression. A particularly interesting parameter is total FRAP, which—at the level of over 17.92 μM/mg protein in SWS—differentiates patients with weak/moderate renal failure from those with severe CKD (AUC = 1, sensitivity = 100%, specificity = 100%). However, further studies in a larger population of patients are needed to confirm the clinical usefulness of salivary FRAP.

In view of the constant increase in CKD incidence and high costs of therapy, salivary redox biomarkers may serve as the basis for developing laboratory tests that would enable early, non-invasive diagnosis of chronic kidney disease in children. The FRAP method is cheap and fast, characterized by high repeatability and reproducibility, and—due to the presence of commonly available commercial diagnostic kits—the results of tests can be compared with each other [23].

However, despite the undeniable advantages, salivary redox biomarkers have certain limitations. Diseases of the oral cavity and periodontium may affect FRAP values in saliva. What is more, such factors as medicines taken (e.g., iron, angiotensin converting enzyme inhibitors (ACE-I), angiotensin receptor blocker (ARB)), environmental factors (diet, xenobiotics) and other systemic diseases of proven oxidative stress aetiology (e.g., diabetes, heart failure, thyroid and autoimmune diseases) may also disturb total antioxidant potential.

Summarising, salivary total FRAP, FRAP-UA and UA can be useful for monitoring CKD progression in children. These biomarkers increase with the severity of chronic kidney disease and their concentration in stimulated saliva reflects their content in urine. However, special attention should be paid to salivary total FRAP which distinguishes children with mildly to moderately decreased kidney function from those with severe renal impairment. Further studies are needed to confirm the diagnostic value of salivary FRAP in a larger population of CKD children.

## Figures and Tables

**Table 1:** Clinical characteristics of children with different stages of chronic kidney disease (CKD) and healthy controls.

	Control *n* = 30	Stage 1 *n* = 5	Stage 2 *n* = 8	Stage 3 *n* = 7	Stage 4 *n* = 5	Stage 5 *n* = 5	CKD *n* = 30
Weight (kg)	42.1 ± 6.2	46.06 ± 4.74	51.64 ± 8.36	33.86 ± 4.27	42.34 ± 13.55	32.3 ±7.17	41.79 ± 3.65
Height (cm)	138.2 ± 8.3	145.6 ± 7.66	151.7 ± 5.58	134.4 ± 5.63	143.4 ± 7.34	130.2 ± 10.16	141.7 ± 3.29
Age (yrs)	12.5 ± 0.8	14.35 ± 1.51	13.89 ± 1.3	11.71 ± 1.2	13.35 ± 1.26	11.28 ± 2.04	12.93 ± 0.64
Age of diagnosis (yrs)	ND	12.75 ± 1.23	9.53 ± 2.13	7.82 ± 1.43	6.55 ± 2.55	7.06 ± 1.42	8.76 ± 0.88
eGFR (mL/min/1.73 m^2^)	137.2 ± 8.9	136.6 ± 15.88	75.26 ± 5.65	42.19 ± 5.25	21.76 ± 3.28	12.46 ± 0.83	58.38 ± 8.31
Serum Cr (mg/dL)	0.42 ± 0.1	0.82 ± 0.1	1.07 ± 0.11	1.72 ± 0.19	3.77 ± 0.13	6.62 ± 0.49	2.55 ± 0.39
Serum urea (mg/dL)	18.1 ± 3.4	26.8 ± 7.33	44.63 ± 5.89	57.29 ± 8.23	110.1 ± 9.52	156.2 ± 13.99	74.12 ± 9.09
Proteinuria (mg/24 h)	62.1 ± 3.7	91.5 ± 4.33	490.5 ± 224.5	607 ±329.6	804.3 ± 285.5	794 ± 553.5	561.9 ± 137.3
Albuminuria (mg/24 h)	8 ± 0.9	9.54 ± 0.41	54.53 ± 27.58	124.7 ± 113.8	335.5 ± 96.98	1454 ± 110.2	348 ± 126.4
Ca^2+^ (mmol/L)	ND	2.49 ± 0.04	2.38 ± 0.07	2.39 ± 0.06	2.37 ± 0.01	2.48 ± 0.08	2.42 ± 0.03
Vitamin D_3_ (ng/mL)	ND	24.1 ± 3.33	17.38 ± 3.57	23.86 ± 7.43	22.13 ± 6.56	10.8 ± 3.09	19.71 ± 2.4
PTH (pg/mL)	35.1 ± 5.5	39.03 ± 8.9	54.96 ± 11.58	73.67 ± 16.28	161.4 ± 132.1	703.6 ± 177.5	194.6 ± 60.6
ALP (U/L)	38.5 ± 8.7	109.3 ± 31	196.6 ± 41.31	197.3 ± 23.24	239.7 ± 89.27	372.6 ± 192.6	223.8 ± 41.33
Hgb (g/dL)	14.8 ± 0.3	13.64 ± 0.75	13.39 ± 0.82	12.14 ± 0.72	10.43 ± 0.21	11.02 ± 0.97	12.31 ± 0.4
Hct (%)	39.8 ± 1	39.88 ± 1.95	38.33 ± 2.01	35.31 ± 1.71	31.24 ± 0.27	32.44 ± 2.58	35.72 ± 0.99
Fe (μg/dL)	80.2 ± 2	55.2 ± 10.44	78.13 ± 11.85	67.14 ± 8.85	82.2 ± 2.46	106.4 ± 16.16	77.13 ± 5.57
Ferritin (ng/mL)	ND	53.23 ± 13.17	78.89 ± 28.41	106.5 ± 41.02	183.5 ± 66.46	320.6 ± 66.52	138.8 ± 25.01
SBP (mmHg)	107 ± 1.5	116 ± 6.3	121.8 ± 6.34	113.4 ± 3.77	111.9 ± 1.21	119.4 ± 6.19	116.8 ± 2.37
DBP (mmHg)	65 ± 2.6	70.8 ± 3.12	68 ± 3.08	69.57 ± 2.46	74.27 ± 1.17	79.6 ± 5.01	71.81 ± 1.52
Hypertension n (%)	0 (0)	1 (20)	1 (12.5)	1 (14.3)	2 (40)	3 (60)	8 (26.7)
Dialysis n (%)	0 (0)	0 (0)	0 (0)	0 (0)	1 (20)	5 (100)	6 (20)
Drugs < 5 per day n (%)	0 (0)	2 (40)	4 (50)	5 (71.4)	2 (40)	1 (20)	14 (46.7)
Drugs ≥ 5 per day n (%)	0 (0)	3 (60)	4 (50)	2 (28.6)	3 (60)	4 (80)	16 (53.3)

ALP—alkaline phosphatase; Ca^2+^—calcium ions; DBP—diastolic blood pressure; eGFR—estimated glomerular filtration rate using Schwartz formula; Fe—serum iron levels; Hgb—haemoglobin level; Hct—haematocrit; PTH—parathyroid hormone; SBP—systolic blood pressure; sCr—serum creatinine.

**Table 2:** Salivary gland function and dental examination of children at different stages of chronic kidney disease (CKD) and healthy controls.

	Control *n* = 30	Stage 1 *n* = 5	Stage 2 *n* = 8	Stage 3 *n* = 7	Stage 4 *n* = 5	Stage 5 *n* = 5	CKD *n* = 30	ANOVA *p*
Total protein NWS (μg/mL)	1371 ± 78.52	832.2 ± 31.45 *	976 ± 46.75 *	931.1 ± 89.26 *	919.5 ± 52.16	897.7 ± 72.63 *	918.4 ± 29.07 *	<0.001
Total protein SWS (μg/mL)	1308 ± 30.77	907.1 ± 89.52 *	916.3 ± 64.24 *	1031 ± 64.17 *	998.9 ± 40.09 *	890.1 ± 78.15 *	950.9 ± 30.56 *	<0.001
NWS flow rate (mL/min)	0.56 ± 0.02	0.30 ± 0.03 *	0.34 ± 0.04 *	0.33 ± 0.03 *	0.24 ± 0.05 *	0.31 ± 0.08 *	0.31 ± 0.02 *	<0.001
SWS flow rate (mL/min)	1.42 ± 0.05 *	0.85 ± 0.09 *	0.78 ± 0.1 *	0.76 ± 0.1 *	0.81 ± 0.12 *	1.09 ± 0.08	0.84 ± 0.05 *	<0.001
DMFT	3.2 ± 0.5	3.1 ± 0.4	3.0 ± 0.7	3.2 ± 0.3	3.6 ± 0.8	3.5 ± 0.5	3.3 ± 0.5	NS
dmft	10.1 ± 0.5	10.8 ± 0.2	11 ± 0.2	12.5 ± 0.3	11.1 ± 0.5	12.4 ± 0.8	11.5 ± 0.4	NS
PBI	0 ± 0.1	0 ± 0.1	0 ± 0.3	0 ± 0.3	0 ± 0.3	0 ± 0.3	0 ± 0.3	NS
GI	0 ± 0.2	0 ± 0.2	0 ± 01	0 ± 0.2	0 ± 0.2	0 ± 0.2	0 ± 0.2	NS

DMFT—decay, missing, filled teeth; GI—Gingival Index; NWS—non-stimulated whole saliva; SWS—stimulated whole saliva. * *p* < 0.05 vs. the control group.

**Table 3:** Receiver operating characteristic (ROC) analysis of total antioxidant capacity measured by FRAP in saliva, plasma and urine of children with chronic kidney disease (CKD) and healthy controls.

	AUC	*p*-Value	Cut-Off	Confidence Intervals	Sensitivity (%)	Specificity (%)
*NWS*						
Total FRAP (μM/mg protein)	0.7	0.008	>7.90	0.56–0.84	73.33	73.33
FRAP-UA (μM/mg protein)	0.81	<0.001	>3.68	0.69–0.93	80	80
UA (μM/mg protein)	0.54	NS	>3.84	0.39–0.69	53.33	43.33
ΔFRAP (%)	0.62	NS	<53.76	0.47–0.76	60	60
*SWS*						
Total FRAP (μM/mg protein)	0.95	<0.001	<10.41	0.9–1.01	90	90
FRAP-UA (μM/mg protein)	0.95	<0.001	>3.92	0.9–1.02	93.33	90
UA (μM/mg protein)	0.94	<0.001	>6.79	0.9–1	90	90
ΔFRAP (%)	0.76	<0.001	>62.2	0.64–0.89	70	73.33
*Plasma*						
Total FRAP (μM/mg protein)	0.69	0.01	>5.78	0.55–0.83	66.67	70
FRAP-UA (μM/mg protein)	0.57	NS	>1.29	0.42–0.72	60	53.33
UA (μM/mg protein)	0.72	0.003	>4.35	0.59–0.86	70	70
ΔFRAP (%)	0.63	NS	>77.53	0.48–0.78	56.67	60
*Urine*						
Total FRAP (μM/mg protein)	0.98	<0.001	>10.46	0.96–1.01	90	90
FRAP-UA (μM/mg protein)	0.95	<0.001	>4.51	0.89–1.01	86.67	90
UA (μM/mg protein)	0.98	<0.001	>6.72	0.95–1.01	90	90
ΔFRAP (%)	0.8	<0.001	>61.41	0.67–0.92	80	80

FRAP—ferric ion reducing antioxidant parameter; FRAP-UA—UA-independent FRAP; NS—no significance; NWS—non-stimulated whole saliva; SWS—stimulated whole saliva; UA—uric acid.

**Table 4:** Receiver operating characteristic (ROC) analysis of total antioxidant capacity measured by FRAP in saliva, plasma and urine of children with stage 1–3 and 4–5 of chronic kidney disease (CKD).

	AUC	*p*-Value	Cut-Off	Confidence Intervals	Sensitivity (%)	Specificity (%)
*NWS*						
Total FRAP (μM/mg protein)	0.61	NS	<9.28	0.4–0.81	58.33	55
FRAP-UA μM/mg protein)	0.55	NS	>5.02	0.31–0.78	50	50
UA (μM/mg protein)	0.53	NS	<4.32	0.3–0.76	60	55
ΔFRAP (%)	0.61	NS	<44.77	0.39–0.83	60	60
*SWS*						
Total FRAP (μM/mg protein)	1.00	<0.001	>17.92	1–1	100	100
FRAP-UA μM/mg protein)	0.91	<0.001	>5.932	0.8–1.01	80	75
UA (μM/mg protein)	0.96	<0.001	>12.07	0.9–1.02	90	90
ΔFRAP (%)	0.55	NS	>66.32	0.34–0.77	60	55
*Plasma*						
Total FRAP (μM/mg protein)	0.63	NS	>6.43	0.4–0.86	60	65
FRAP-UA (μM/mg protein)	0.61	NS	>1.41	0.38–0.83	60	55
UA (μM/mg protein)	0.62	NS	>4.97	0.4–0.85	60	60
ΔFRAP (%)	0.56	NS	<77.98	0.34–0.78	50	55
*Urine*						
Total FRAP (μM/mg protein)	0.99	<0.001	>20	0.96–1.02	100	95
FRAP-UA (μM/mg protein)	0.89	<0.001	>6.83	0.77–1	80	75
UA (μM/mg protein)	1	<0.001	>12.95	1–1	100	100
ΔFRAP (%)	0.73	0.047	>65.16	0.52–0.93	70	70

FRAP—ferric ion reducing antioxidant parameter; FRAP-UA—UA-independent FRAP; NS—no significance; NWS—non-stimulated whole saliva; SWS—stimulated whole saliva; UA—uric acid.

**Table 5:** Correlations between salivary and plasma/urine total antioxidant capacity measured by FRAP in children with chronic kidney disease (CKD).

	Total FRAP plasma	FRAP-UA Plasma	UA Plasma	ΔFRAP Plasma	Total FRAP Urine	FRAP-UA Urine	UA Urine	ΔFRAP Urine
Total FRAP NWS	−0.12	−0.42 *	−0.01	0.28	0.01	−0.05	0.04	0.17
FRAP-UA NWS	−0.12	−0.32	−0.03	0.19	0.09	0.01	0.13	0.25
UA NWS	−0.10	−0.40 *	0.01	0.29	−0.04	−0.08	−0.02	0.1
ΔFRAP NWS	0.05	−0.16	0.10	0.19	−0.16	−0.14	−0.16	−0.09
Total FRAP SWS	0.26	0.45 *	0.16	−0.20	0.94 *	0.83 *	0.93 *	0.21
FRAP-UA SWS	−0.01	0.19	−0.07	−0.18	0.72 *	0.65 *	0.71 *	0.14
UA SWS	0.36 *	0.52 *	0.25	−0.19	0.93 *	0.84 *	0.93 *	0.22
ΔFRAP SWS	0.44 *	0.50 *	0.34	−0.13	0.47 *	0.44 *	0.47 *	0.15

FRAP—ferric ion reducing antioxidant parameter; FRAP-UA—UA-independent FRAP; NWS—non-stimulated whole saliva; SWS—stimulated whole saliva; UA—uric acid. * *p* < 0.05.

**Table 6:** Correlations between total antioxidant capacity measured by FRAP and clinical characteristics of children with chronic kidney disease (CKD).

	Total FRAP NWS	FRAP-UA NWS	UA NWS	ΔFRAP NWS	Total FRAP SWS	FRAP-UA SWS	UA SWS	ΔFRAP SWS
Weight (kg)	0.02	−0.04	0.05	−0.03	−0.2	−0.12	−0.21	−0.14
Height (cm)	0.23	0.10	0.27	0.10	−0.26	−0.16	−0.28	−0.19
Age (yrs)	0.23	0.18	0.23	−0.01	−0.16	−0.11	−0.17	−0.14
Age of diagnosis (yrs)	0.17	0.03	0.23	0.16	−0.37 *	−0.23	−0.39 *	−0.29
eGFR (mL/min/1.73 m^2^)	−0.05	−0.16	0.03	0.18	−0.83 *	−0.72 *	−0.79 *	−0.28
sCr (mg/dL)	0.10	0.19	0.04	−0.09	0.78 *	0.79 *	0.69 *	0.09
Serum urea (mg/dL)	0.02	0.14	−0.05	−0.12	0.73 *	0.70 *	0.67 *	0.14
Proteinuria (mg/24 h)	−0.17	−0.04	−0.22	−0.30	0.49 *	0.36	0.50 *	0.25
Albuminuria (mg/24 h)	0.08	0.14	0.03	−0.02	0.74 *	0.74 *	0.66 *	0.11
Ca^2+^ (mmol/L)	0.31	0.13	0.36	0.39 *	−0.07	−0.03	−0.09	−0.06
Vitamin D_3_ (ng/mL)	0.04	0.04	0.03	0.01	−0.18	−0.13	−0.19	−0.07
PTH (pg/mL)	0.13	0.31	0.01	−0.19	0.65 *	0.72 *	0.55 *	−0.01
ALP (U/L)	−0.06	0.21	−0.19	−0.29	0.49 *	0.38	0.49 *	0.22
Hgb (g/dL)	0.27	0.09	0.33	0.38 *	−0.48 *	−0.44 *	−0.45 *	−0.06
Hct (%)	0.29	0.06	0.38 *	0.45 *	−0.53 *	−0.48 *	−0.50 *	−0.12
Fe (μg/dL)	−0.18	0.04	−0.27	−0.28	0.46 *	0.34 *	0.47 *	0.28
Ferritin (ng/mL)	0.13	0.27	0.03	−0.17	0.53 *	0.65 *	0.42 *	−0.09
SBP (mmHg)	−0.12	−0.19	−0.06	0.06	−0.03	0.01	−0.04	−0.04
DBP (mmHg)	−0.13	−0.17	−0.09	0.05	0.23	0.23	0.20	0.04

FRAP—ferric ion reducing antioxidant parameter; FRAP-UA—UA-independent FRAP; NWS—non-stimulated whole saliva; SWS—stimulated whole saliva; UA—uric acid. * *p* < 0.05.

## References

[1] Levey A.S., De Jong P.E., Coresh J., Nahas M.E., Astor B.C., Matsushita K., Gansevoort R.T., Kasiske B.L., Eckardt K.U. (2011). The definition, classification, and prognosis of chronic kidney disease: A KDIGO Controversies Conference report. Kidney Int..

[2] Becherucci F., Roperto R.M., Materassi M., Romagnani P. (2016). Chronic kidney disease in children. Clin. Kidney J..

[3] Koye D.N., Magliano D.J., Nelson R.G., Pavkov M.E. (2018). The Global Epidemiology of Diabetes and Kidney Disease. Adv. Chronic Kidney Dis..

[4] Willis K., Cheung M., Slifer S. (2013). KDIGO 2012 Clinical Practice Guideline for Evaluation & Management of CKD. Kidney Int..

[5] Lopez-Giacoman S. (2015). Biomarkers in chronic kidney disease, from kidney function to kidney damage. World J. Nephrol..

[6] Modaresi A., Nafar M., Sahraei Z. (2015). Oxidative stress in chronic kidney disease. Iran. J. Kidney Dis..

[7] Nakanishi T., Kuragano T., Nanami M., Nagasawa Y., Hasuike Y. (2018). Misdistribution of Iron and Oxidative Stress in Chronic Kidney Disease. Free Radic. Biol. Med..

[8] Putri A.Y., Thaha M. (2014). Role of Oxidative Stress on Chronic Kidney Disease Progression. Acta Med. Indones..

[9] Kuchta A., Pacanis A., Kortas-Stempak B., Ćwiklińska A., Ziȩtkiewicz M., Renke M., Rutkowski B. (2011). Estimation of oxidative stress markers in chronic kidney disease. Kidney Blood Press. Res..

[10] Schei J., Fuskevåg O.M., Stefansson V.T.N., Solbu M.D., Jenssen T.G., Eriksen B.O., Melsom T. (2018). Urinary Markers of Oxidative Stress are Associated with Albuminuria but not GFR Decline. Kidney Int. Rep..

[11] Maciejczyk M., Szulimowska J., Skutnik A., Taranta-Janusz K., Wasilewska A., Wiśniewska N., Zalewska A. (2018). Salivary Biomarkers of Oxidative Stress in Children with Chronic Kidney Disease. J. Clin. Med..

[12] Lakshmi B.S., Devi N.H., Suchitra M.M., Rao P.V.L.N.S., Kumar V.S. (2018). Changes in the inflammatory and oxidative stress markers during a single hemodialysis session in patients with chronic kidney disease. Ren. Fail..

[13] Javaid M.A., Ahmed A.S., Durand R., Tran S.D. (2016). Saliva as a diagnostic tool for oral and systemic diseases. J. Oral Biol. Craniofacial Res..

[14] Maciejczyk M., Skutnik-Radziszewska A., Zieniewska I., Matczuk J., Domel E., Waszkiel D., Żendzian-Piotrowska M., Szarmach I., Zalewska A. (2019). Antioxidant Defense, Oxidative Modification, and Salivary Gland Function in an Early Phase of Cerulein Pancreatitis. Oxid. Med. Cell. Longev..

[15] Knaś M., Maciejczyk M., Waszkiel D., Zalewska A. (2013). Oxidative stress and salivary antioxidants. Dent. Med. Probl..

[16] Al-Rawi N.H. (2011). Oxidative stress, antioxidant status and lipid profile in the saliva of type 2 diabetics. Diabetes Vasc. Dis. Res..

[17] Nair A., Nair B. (2017). Comparative analysis of the oxidative stress and antioxidant status in type II diabetics and nondiabetics: A biochemical study. J. Oral Maxillofac. Pathol..

[18] Fejfer K., Buczko P., Niczyporuk M., Ładny J.R., Hady H.R., Knaś M., Waszkiel D., Klimiuk A., Zalewska A., Maciejczyk M. (2017). Oxidative Modification of Biomolecules in the Nonstimulated and Stimulated Saliva of Patients with Morbid Obesity Treated with Bariatric Surgery. Biomed Res. Int..

[19] Knaś M., Maciejczyk M., Sawicka K., Hady H.R., Niczyporuk M., Ładny J.R., Matczuk J., Waszkiel D., Żendzian-Piotrowska M., Zalewska A. (2016). Impact of morbid obesity and bariatric surgery on antioxidant/oxidant balance of the unstimulated and stimulated human saliva. J. Oral Pathol. Med..

[20] Kułak-Bejda A., Waszkiewicz N., Bejda G., Zalewska A., Maciejczyk M. (2019). Diagnostic Value of Salivary Markers in Neuropsychiatric Disorders. Dis. Markers.

[21] Klimiuk A., Maciejczyk M., Choromańska M., Fejfer K., Waszkiewicz N., Zalewska A. (2019). Salivary Redox Biomarkers in Different Stages of Dementia Severity. J. Clin. Med..

[22] Bibi G., Green Y., Nagler R.M. (2008). Compositional and oxidative analysis in the saliva and serum of predialysis chronic kidney disease patients and end-stage renal failure patients on peritoneal dialysis. Ther. Apher. Dial..

[23] Duplancic D., Kukoc-Modun L., Modun D., Radic N. (2011). Simple and rapid method for the determination of uric acid-independent antioxidant capacity. Molecules.

[24] Schwartz G.J., Muñoz A., Schneider M.F., Mak R.H., Kaskel F., Warady B.A. (2009). New Equations to Estimate GFR in Children with CKD. J. Am. Soc. Nephrol..

[25] National Institutes Health (NIH) (2005). The Fourth Report on the Diagnosis, Evaluation, and Treatment of High Blood Pressure in Children and Adolescents.

[26] National Kidney Foundation (2002). K/DOQI clinical practice guidelines for chronic kidney disease: Evaluation, classification, and stratification. Am. J. Kidney Dis..

[27] Choromańska M., Klimiuk A., Kostecka-Sochoń P., Wilczyńska K., Kwiatkowski M., Okuniewska N., Waszkiewicz N., Zalewska A., Maciejczyk M. (2017). Antioxidant defence, oxidative stress and oxidative damage in saliva, plasma and erythrocytes of dementia patients. Can salivary AGE be a marker of dementia?. Int. J. Mol. Sci..

[28] World Health Organization (2013). Oral Health Surveys: Basic Methods.

[29] Lushchak V.I. (2014). Free radicals, reactive oxygen species, oxidative stress and its classification. Chem. Biol. Interact..

[30] Żukowski P., Maciejczyk M., Waszkiel D. (2018). Sources of free radicals and oxidative stress in the oral cavity. Arch. Oral Biol..

[31] Nagler R.M., Klein I., Zarzhevsky N., Drigues N., Reznick A.Z. (2002). Characterization of the differentiated antioxidant profile of human saliva. Free Radic. Biol. Med..

[32] Maciejczyk M., Matczuk J., Żendzian-Piotrowska M., Niklińska W., Fejfer K., Szarmach I., Ładny J.R., Zieniewska I., Zalewska A. (2018). Eight-Week Consumption of High-Sucrose Diet Has a Pro-Oxidant Effect and Alters the Function of the Salivary Glands of Rats. Nutrients.

[33] Feinstein H., Schramm M. (1970). Energy Production in Rat Parotid Gland: Relation to Enzyme Secretion and Effects of Calcium. Eur. J. Biochem..

[34] Zalewska A., Ziembicka D., Żendzian-Piotrowska M., Maciejczyk M. (2019). The Impact of High-Fat Diet on Mitochondrial Function, Free Radical Production, and Nitrosative Stress in the Salivary Glands of Wistar Rats. Oxid. Med. Cell. Longev..

[35] Johnson D.W., Jones G.R.D., Mathew T.H., Ludlow M.J., Chadban S.J., Usherwood T., Polkinghorne K., Colagiuri S., Jerums G., MacIsaac R. (2012). Chronic kidney disease and measurement of albuminuria or proteinuria: A position statement. Med. J. Aust..

